# Adopting a Global Safety Standard for the Prevention of Ebola Needle-Stick Exposures

**DOI:** 10.1017/ice.2015.62

**Published:** 2015-04-01

**Authors:** Gabriella De Carli, Francesco M. Fusco, Vincenzo Puro, Giuseppe Ippolito

**Affiliations:** 1 Department of Epidemiology and Pre-Clinical Research, National Institute for Infectious Diseases L. Spallanzani-IRCCS, Rome, Italy. Members of the EuroNHID Working Group: Brodt RH, Schilling S, Gottschalk R, Maltezou HC, Bannister B, Vetter N, Kojouharova M, Parmakova K, Skinhoej P, Kronborg G, Siikamaki H, Brouqui P, Perronne C, Lambert J, Hemmer R, Borg M, Azzopardi CM, Brantsæter AB, Fjellet AL, Horban A, Strle F, Trilla A, De Iaco G.

*To the Editor*—As of February 2, 2015, 13 healthcare workers (HCWs) working for international organizations or volunteers who acquired Ebola Virus Disease (EVD) in West Africa have been medically evacuated, and another 12 were repatriated after sustaining a significant exposure (frequently a contaminated needle stick)^1^ ^,^ ^2^ to specialized isolation facilities in Europe with high-level isolation units.

These isolation facilities were identified early in the course of the current epidemic as those better equipped and prepared to safely manage EVD cases, being able to minimize the occupational risk for HCWs.

Actually, in 2009 the European Network for Highly Infectious Diseases project,^3^ aiming to enhance and maintain preparedness and response, cooperation, and exchange of information and experiences between isolation facilities identified by the national health authorities as referral centers in Europe, already recognized that HCWs had not only been extensively trained in adopting high isolation precautions but had also been adequately resourced with safety-engineered devices integrating a protective mechanism for needle-stick prevention. Adoption of safety-engineered devices at that time was not required by European regulation, being implemented on a voluntary basis by hospitals and facilities. All 48 isolation facilities visited had in place a written procedure for needle-stick prevention and post-exposure management as well as standardized systems for injury recording. All had adopted at least 1 safety-engineered device (Table 1), all hollow-bore devices, mainly for blood collection or central and peripheral vascular access. In 23 facilities, 7 or more of these devices were routinely in use, 3–6 were in use in 22 facilities, and <3 were in use in the remaining 3 facilities. In 46 facilities, some strategies were in place for the promotion and implementation of safe practices for needle-stick prevention, with 25 performing specific practical exercises. Actions to monitor the correct application of preventive procedures were specified by 18 facilities: 11 performed specific audits on this issue; HCWs were monitored by an expert in 4 facilities; practical test after training and examination of sharp containers were performed in 1 facility each. Finally, in 3 facilities the only monitoring strategy was represented by the analysis of accidents.

**TABLE 1:** Safety-Engineered Devices in Use in 48 Isolation Facilities Designated for the Referral and Management of Highly Infectious Diseases in 16 European Countries (EuroNHID), 2009–2010

Device	No. (%)
Winged steel needle (butterfly) blood collection sets	37 (77.1)
Hypodermic needles and syringes (sliding sheath/sleeve; needle guards)	30 (62.5)
Shielded or retracting peripheral intravenous catheters	28 (58.3)
Retractable needles/syringes	27 (56.2)
Prefilled syringes	23 (47.9)
Arterial blood gas syringes	22 (45.8)
Safety engineered blood collection needles	22 (45.8)
Safety engineered blood collection needles with tube holders	22 (45.8)
Needleless jet injection systems	17 (35.4)
Needleless intravenous access – blunted cannulas	17 (35.4)
Peripherally inserted central catheter kit with integral needle protection	13 (27.1)
Prefilled medication cartridge with safety needles	12 (25.0)
Central venous catheter kit with integral needle protection	11 (22.9)
Epidural/spinal needles with safety epidural needle	6 (12.5)

This extensive preparation and training is necessary because performing an invasive procedure normally represents a risk and this risk is higher when wearing a full gear with goggles and facial respirators. Among specific factors possibly increasing the risk of contaminated sharps injuries, visibility, communication, and range of motion were found to affect this risk. High-efficiency particulate air respirators have a negative impact on each of these variables,^4^ and wearing personal protective equipment for highly infectious diseases may, to a degree, hinder a healthcare worker's ability to perform routine tasks.

Recent debate among medical experts is sharply divided over whether most patients in West Africa should, or can, be given intravenous hydration. Some have argued that more aggressive treatment with IV fluids is medically possible and a moral obligation, and others have counseled caution, saying that pushing too hard would put overworked doctors and nurses in danger. It is difficult to insert needles while wearing three pairs of gloves and foggy goggles: IVs must be monitored; drawing virus-laden blood for tests is dangerous; and patients sometimes yank needles out.^5^ Indeed, needle-stick injuries have been responsible for transmitting Ebola and other hemorrhagic fever viruses in past outbreaks,^6^ and needle sticks are considered a ‘high risk’ epidemiologic factor when evaluating a person for exposure to Ebola virus. Among the HCWs repatriated from Sierra Leone, a South Korean doctor was evacuated to Germany after sustaining a needle stick while using a hypodermic needle to draw blood from a patient with a very high viral load who died the next day from EVD. After sustaining a needle stick with a needle left in a bottle because the sharps container was too full, a US doctor was medically evacuated, transported in isolation to the National Institutes of Health in Bethesda, Maryland, and treated with an experimental drug to prevent EVD; eventually it was determined that he was uninfected. For the management of HCWs returning from Ebola-affected areas, the European Centre for Disease Prevention and Control has recommended active monitoring and restricted movement, social interactions, and engagement in clinical activities for those HCWs who sustain needle sticks.^7^

The first recommendation by the Emergency Care Research Institute (ECRI) regarding equipment-related preparedness for Ebola is to use needle-stick prevention devices, to ensure that all areas likely to be used for treating Ebola patients have a full complement of those devices, and to try out available devices to make sure their protective features can be safely engaged when staff are double- or triple-gloved.^8^

HCW health and safety are of great concern in Europe and in the United States, and they should be even more a priority in epidemic areas. Where safety devices were introduced, with the help and support of international programs for patient and HCW safety, no further needle-stick injuries were reported in relation to the specific at-risk procedure.^9^ International health organizations and authorities should support the provision of protected needles to all those who are working in the worst affected West African countries. Decreasing HCW risk should also be a moral obligation to help create a “safe working environment to foster the development of local expertise,”^10^ as well as to protect foreign HCWs seeking to stem the Ebola epidemic. Such measures include more safely performing invasive procedures such as intravenous rehydration and constant measuring of blood chemistry, which hopefully increase patient survival, and avoiding preventable injuries.
